# British Parliament

**Published:** 1848-02

**Authors:** 


*British Parliament.—Evidence taken before the Select Committee on Medical Legislation.*

*Sir Benjamin Brodie on Free Trade in Medicine.*—My own opinion is, that the profession would do better if no one could be prosecuted merely for engaging in private practice, and that the only restriction should be that persons who had not licenses should not be eligible for public institutions or public appointments. I would endeavour to affect the great object to be secured for the public, of obtaining the best acquirements, not by penalties for the want of them, but by encouraging the possession of them. I think that in the same way as individuals are always better from having to depend upon their own conduct and character, and not being propped up by others, so the profession would be better from having to depend upon its own character, and not being protected by penalties. I remember the bills of 1845. They proceed upon that principle of holding out inducements, and not inflicting penalties, and I entirely approved of them. In that respect those bills effected what I conceive to be politic, and many of the profession think the same: but the great mass differ from me in opinion. They would desire the infliction of severe penalties for practising without due legal authority. I would not recommend that course.

The apothecaries have a very strict monopoly: that is, their act of Parliament gives them a very strict monopoly, but they cannot act upon the act of Parliament; they prosecute a few persons, but the great number of persons who practise without their license are not prosecuted. Penalties, I may say, do not interfere with the practice of quacks or really uneducated persons at all. There will always be a tendency to employ quacks, because all must die in their turn. The medical profession can never do all that is required of them to do, and those who cannot be relieved by the regular craft will of course naturally look to others. While the demand for empirical practice is so general, I suspect that the proceeding by penalty to check it will be ineffectual. It is very desirable to stop it, if possible, but I do not think it can be done. Looking to the state of the profession generally, under the law as it now exists, and considering the bill for the alteration of the law as proposed in the present session, I would rather leave the profession as it is.

At the same time I am of opinion that the law regulating the medical profession is very susceptible of improvement in some respects.

I think it a very great hardship that no one should be admitted to general practice here who has not been authorized by the Apothecaries' Society; I think it a hardship that those who are educated in Scotland or Ireland, who, from some technicality, cannot be examined at Apothecaries' Hall, should not be permitted to practise in England; but I think that the object may be attained by some other and better means than by that bill. Any man may practise as a surgeon in England without subjecting himself to a penalty; and I do not think that surgery has at all suffered from that cause. I think surgery has more advanced than any other part of the profession, though there is no legal penalty upon parties practising as surgeons without a license. I think that the profession might, without much harm, be thrown open in the same manner as surgery is; but the view which I take is this: when a person gets an opinion about his own case, he has a right to consult anybody, and anybody should have a right to attend him; but where one person has to appoint another to attend a third person, he should be required to appoint somebody who has a regular license to practice. Therein consists the difference between the appointment by public bodies and the Government, and the choice of an individual, with respect to his own medical attendant; and that would extend even to schools and workhouses and ships. Wherever third parties appoint medical advisers, there the law should step in and obtain some security with respect to the competency of the party so to be appointed. That is the line that I would draw. Each individual should, according to law, exercise his own free choice, but public bodies should be compelled by law to employ only persons whose skill had been tested by some public authority. I think that would be a very good system. I do not say that it would be practicable to introduce it with the present feelings of the profession; but I very much suspect that if you wait twenty years the profession generally will be of the same opinion with myself.—*Lond. Med. Times.*

